# Sensitivity Analysis of *Pyrenophora tritici-repentis* to Quinone-Outside Inhibitor and 14α-Demethylase Inhibitor Fungicides in Latvia

**DOI:** 10.3390/pathogens13121060

**Published:** 2024-12-02

**Authors:** Jānis Kaņeps, Biruta Bankina, Inga Moročko-Bičevska, Katrīna Apsīte, Ance Roga, Dāvids Fridmanis

**Affiliations:** 1 Faculty of Agriculture and Food Technology, Latvia University of Life Sciences and Technologies, Liela Street 2, LV-3001 Jelgava, Latvia; 2 Institute of Horticulture, Latvia University of Life Sciences and Technologies, Graudu Street 1, LV-3701 Dobele, Latvia; 3 Latvian Biomedical Research and Study Centre, Ratsupites Street 1, k-1, LV-1067 Riga, Latvia

**Keywords:** tan spot, wheat diseases, prothioconazole, mefentrifluconazole, pyraclostrobin, azoxystrobin

## Abstract

Tan spot caused by *Pyrenophora tritici-repentis* is a severe threat to wheat production in all major wheat-growing regions. Sustainable tan spot control can be achieved by an integrated approach, including responsible management of fungicide sprays. The data about the sensitivity of *P. tritici-repentis* to various fungicides in the Baltic Sea region are rare. In this study, we described the variation of *P. tritici-repentis* sensitivity to four fungicide active ingredients to detect the formation of resistance to the most commonly used quinone-outside inhibitor (QoI) and 14α-demethylase inhibitor (DMI) fungicides in the pathogen’s population in Latvia. The effect of prothioconazole, mefentrifluconazole, pyraclostrobin, and azoxystrobin on 93 *P. tritici-repentis* strains from various hosts was tested in vitro by assessing mycelium linear growth inhibition at three different active ingredient concentrations (0 0.01, 0.1 and 0.5 mg L^−1^). Pathogen sensitivity significantly (*p* < 0.001) varied between the fungicide active ingredients and strains. The prothioconazole (concentration 0.5 mg L^−1^) had the most significant effect, with a median mycelial growth inhibition of 70.34%, followed by pyraclostrobin (47.02%), azoxystrobin (24.24%), and mefentrifluconazole (11.11%). Mutation G143A was detected in *cytb* gene sequences and confirmed the resistance formation in Latvia’s *P. tritici-repentis* population, while F129L and G137R mutations were absent. This study provided insight into *P. tritici-repentis* population’s sensitivity to active ingredients of DMI and OoI fungicide groups, helping to fill the knowledge gap about the pathogen fungicide sensitivity in this region.

## 1. Introduction

Tan spot caused by *Pyrenophora tritici-repentis* is one of the most economically significant wheat diseases in Latvia and in other wheat-growing regions, such as Canada and Pampas in South America [1,2]. Due to the disease, yield losses can reach 10–48% [3,4]. *P. tritici-repentis* is characterized as a diverse pathogen in terms of morphological traits (colony colour, colony texture, etc.), symptom phenotype on various hosts and presence of mobile elements in the genome [5,6,7,8,9]. There is evidence that Europe harbours one of the world’s most diverse *P. tritici-repentis* populations, and it is a major diversity donor for other world regions [10]. However, the European population of *P. tritici-repentis* is still insufficiently described.

The primary inoculum (ascospores) originates from pseudothecia in residues of wheat. During vegetation season, the pathogen spreads with rain- or wind-borne conidia that develops on mature necrotic lesions [11]. The level of tan spot can be decreased via agricultural practices such as soil ploughing and crop rotation [12], but these solutions are starting to lose their relevance in Baltic countries. There are very few field crops in Latvia that can be included in crop rotation and provide the same economic output as wheat [13], which results in an increase in wheat-growing areas. Additionally, EU policies stimulate the adoption of conservation soil tillage technologies that promote overwintering of the pathogen [14,15]. Fungicides play a crucial role in effective tan spot management. *P. tritici-repentis* is mainly controlled by 14α-demethylase inhibitors (DMIs; azoles) and quinone-outside inhibitors (QoIs; strobilurins). Prothioconazole is one of the widely used DMIs in cereal disease control, while mefentrifluconazole is one of the newest additions to triazole fungicides, with excellent results in regards to *Zymoseptoria tritici* control [16]. Similarly, pyraclostrobin and azoxystrobin are QoIs that are highly effective against most plant pathogens and are widely used for cereal disease control [17]. Succinate dehydrogenase inhibitors (SDHIs, carboxamides) are not used in tan spot control due to the low intrinsic activity of the compounds [18]. Two factors largely determine the effectiveness of fungicides: spraying time and the relative effectiveness of the fungicide active ingredients [5,19]. The application time depends on the region’s typical agro-meteorological conditions and the tan spot’s severity. If a fungicide is applied at the optimal time with sufficient spraying quality, the relative sensitivity of the pathogen to the active substances of the fungicide will be the main factor that determines the effectiveness of the disease control.

Jørgensen and Olsen (2007) observed variations in fungicide efficacy active ingredients belonging to the same mode of action [14]. This heterogeneity can be further influenced by fungicide target site mutations in the genome that can occur in the *P. tritici-repentis* population. There are reports that *P. tritici-repentis* has developed target-site mutations to increase resistance to QoIs, but there are no data about resistance against DMIs. Three QoI target-site mutations are known in *P. tritici-repentis*: F129L, G143A and G137R [17,20,21]. Previously, more F129L than G143A mutations have been found in *P. tritici-repentis* strains [17,20]. However, recent reports show that mutation G143A is becoming more common, while F129L is not detected any more [21,22].

The available fungicide active substances become more and more limited, increasing fungicide resistance risks [23]. Subsequently, this problem poses a significant risk to crop production, including wheat, in the European Union (EU). There is a necessity to create and sustain fungicide resistance monitoring programs in the pathogen regional populations. Therefore, the knowledge about the diverse sensitivity of the pathogens to various fungicides in each region is crucial. The aim of this study was to describe the variation of *P. tritici-repentis* strain sensitivity to different fungicide active ingredients and detect the formation of resistance to the most commonly used QoI fungicides in the Latvian *P. tritici-repentis* population.

## 2. Materials and Methods

### 2.1. Fungal Strains

In total, 80 *Pyrenophora tritici-repentis* strains from Latvia were obtained from various hosts, years and geographic locations, (Table 1). The collection was supplemented with one strain from wheat leaf from Lithuania and 12 strains from other collections in the Czech Republic (The Crop Research Institute) and Finland (Natural Resources Institute Finland (LUKE). Strains from the USA and Canada stored in the Crop Research Institute were obtained with the permission of emeritus Prof. Shaukat Ali. The complete list and details of strains included in the study are provided in Supplementary Table S1. The strains of *P. tritici-repentis* were purified via the mycelial tip isolation method. All strains are maintained in the collection as mycelium potato dextrose agar plugs in 10% glycerol solution at −80 °C at the Institute of Soil and Plant Science, Latvia University of Life Sciences and Technologies, and cultured when needed.

### 2.2. Determination of Strain Sensitivity to Fungicides

Agar plugs (5 mm diameter) from two-week-old *P. tritici-repentis* colonies were transferred to Petri dishes containing potato dextrose agar (Scharlau; PDA) enriched with one of four tested fungicides: Curbatur (250 g L^−1^ prothioconazole, PTH; Bayer), Lenvyor (100 g L^−1^ mefentrifluconazole, MFZ; BASF), Comet Pro (200 g L^−1^ pyraclostrobin, PYR; BASF) or Mirador 250 SC (250 g L^−1^ azoxystrobin, AZO; ADAMA). All active ingredients are registered for tan spot control in Latvia [24] and are widely used by Latvian farmers. Strain sensitivity was evaluated by cultivation on the media containing one of four active ingredients (a.i.’s) at the concentrations 0 (control), 0.01, 0.1 and 0.5 mg L^−1^ in the dark at 20 °C for one week in three replicates.

The medium should be amended with salicylhydroxamic acid (SHAM) to eliminate alternative respiration during in vitro sensitivity tests to pyraclostrobin and azoxystrobin, but there is evidence that SHAM can be toxic to mycelial growth of *P. tritici-repentis* [17]. Therefore, a preliminary experiment with six randomly selected strains in four replicates was performed to determine the SHAM concentration that has no effect on the mycelial growth of *P. tritici-repentis.* A stock solution of 0.1 g mL^−1^ SHAM was prepared as described by Patel et al. [25]. The effect of SHAM on mycelial growth was tested in four concentrations (0, 5, 10, 20 and 50 mg L^−1^). The 5 mg L^−1^ SHAM concentration had no significant effect on mycelium growth and this concentration was used when testing the effect of pyraclostrobin and azoxystrobin.

After one week of incubation, each colony diameter was measured in millimetres. Strain sensitivity was expressed as the inhibition (%) of fungus linear growth (I), which was calculated with the *Abbot formula*.

$$I=\frac{\left(C-T\right)}{C}\times 100\%$$

where

*I*—inhibition of fungus linear growth (%);

*C*—average diameter of fungus colony in the control samples;

*T*—diameter of fungus colony in the samples containing a.i.’s.

Five strains from Latvia were randomly selected from different host plants to determine the range of half-maximal effective concentration of fungicide a.i.’s (EC_50_) values within the *P. tritici-repentis* population (Table 2), with the with wild type strain PTR1 included for comparison. The chosen strains were grown in the same conditions as described previously in nine different a.i. concentrations (0 (control), 0.01, 0.1, 0.5, 1, 5, 10, 20, 50 and 100 mg L^−1^) in four replicates. EC_50_ value estimation was performed using R, version 4.3.2 [26] and the ec50estimator package [27].

*P. tritici-repentis* strain sensitivity data distribution was tested with the QQ normal distribution graph, Shapiro–Wilk normality test and Bartlett’s test with software R, version 4.3.2 [26]. Inhibition data were statistically analysed with nonparametric Kruskal–Wallis and Dunn’s post-hoc tests from rstatix and ggbetweenstats packages [28,29]. Pearson correlation coefficients and correlation plots for *P. tritici-repentis* strain sensitivity to different active ingredients were calculated using the ggpairs function from the GGally package [30].

### 2.3. Detection of QoI Target Site Mutations

The possible QoI target site mutations in the *cytb* gene were screened in the same strains used for the EC_50_ value estimation experiment (Table 2). The fungal mycelium was scraped and aseptically collected from actively growing colonies on PDA and ground into a fine powder in liquid nitrogen to extract DNA. DNA extraction was performed with a DNeasy plant mini kit (Qiagen) according to the manufacturer’s guidelines. Two regions of the *cytb* gene were amplified using primers Cytb_F129_F/ Cytb_F129_R and Cytb_G137_G143_F/ Cytb_G137_G143_R using the protocol provided by Sautua and Carmona [21]. The amplification was verified through the inspection of PCR products by 1.2% agarose gel electrophoresis. Prior to sequencing, the PCR products were enzymatically treated with Exonuclease I (0.5 µL) (Thermo Fisher Scientific, Waltham, MA, USA) and Shrimp Alkaline Phosphatase (2 µL) (Thermo Fisher Scientific), incubated for 40 min at 37 °C and inactivated at 95 °C for 20 min to remove the excess of dNTPs and primers. A quantity of 1 µL of purified PCR products was sequenced directly, employing BigDye^®^ Terminator v3.1 Cycle Sequencing reaction mixture (Applied Biosystems, Waltham, MA, USA) and the same primers. Sequencing products were then analysed on a 3130xl Genetic Analyzer (Applied Biosystems).

Sequences were assembled using SeqMan Pro of Lasergene 14 and 17.1 software (DNASTAR Inc., Madison, WI, USA). Obtained sequences were compared with available sequences in GenBank using the Megablast program of NCBI blastn suite (https://blast.ncbi.nlm.nih.gov/Blast.cgi?PROGRAM=blastn, accessed on 20 May 2024). The presence of target site mutations was detected by aligning obtained sequences with *P. tritici-repentis cytb* gene sequences from previous studies available at NCBI GenBank [20,21]. The sequences obtained during this study were deposited in GenBank and are available under accession numbers PP977234-PP977239 for amino acid position F129 and PP977240-PP977245 for amino acid positions G137 and G143.

## 3. Results

### 3.1. Determination of Strain Sensitivity to Fungicides

Among the tested fungicide a.i.’s, there were no significant changes in strain sensitivity patterns at the concentrations of 0.01, 0.1 and 0.5 mg L^−1^. Therefore, the data obtained from the highest concentration (0.5 mg L^−1^) are further described and analysed.

**Figure 1:**
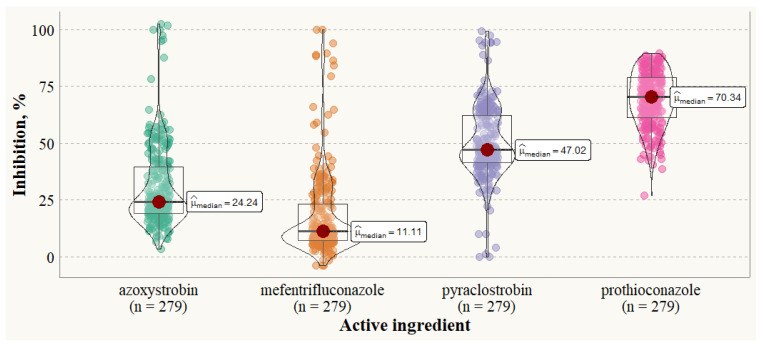
The effect of fungicide active ingredients on mycelial growth of *Pyrenophora tritici-repentis* strains (*p* < 0.001, n = number of data points of calculated inhibition, %, where each strain is represented by three data points).

The sensitivity of *P. tritici-repentis* strains to different fungicide active ingredients at the concentration of 0.5 mg L^−1^ (*p* < 0.001) varied significantly, depending on the active ingredient scale and between the strains (Figure 1). *P. tritici-repentis* strain sensitivity to prothioconazole was uniform, and mycelial growth ranged from 27.08 to 89.61% (median inhibition 70.34%). The sensitivity to other a.i.’s varied greatly, and several strains showed different sensitivity levels compared to the rest of the strains. Only three strains (12015JP, DW2 and DW16) exhibited considerable sensitivity (>80% mycelial growth inhibition) to mefentrifluconazole, while for the rest of *P. tritici-repentis* strains, mycelium growth was inhibited from 0 to 66%. Two strains, 20PTR014 and 20PTR061, had almost no reaction to pyraclostrobin, but the other strains had their mycelial growth inhibited from 20.21 to 99.36%. The efficacy of azoxystrobin ranged from 3.17 to 64.84%, except for a couple of outlaying strains (12015JP, 12016JP and 11020JP), in which mycelium growth was almost completely inhibited.

**Figure 2:**
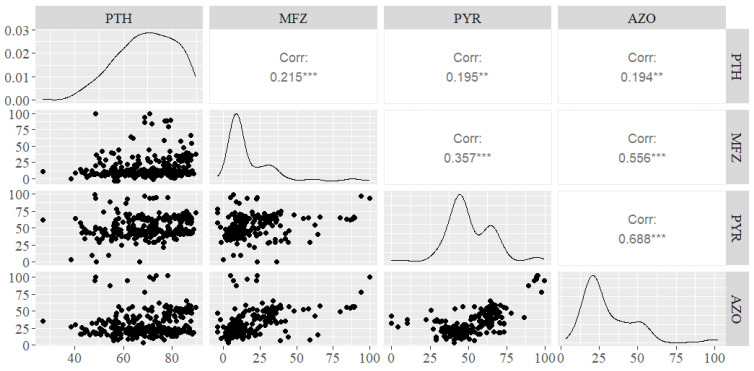
The correlation between fungicide active ingredients regarding *Pyrenophora tritici-repentis* strain growth inhibition. PTH (prothioconazole); MFZ (mefentrifluconazole); PYR (pyraclostrobin); AZO (azoxystrobin). Asterisk symbols annotate significance levels: “***” = 0.001, “**” = 0.01.

A significant correlation (*p* < 0.05) was detected between *P. tritici-repentis* strain inhibition data and all four active ingredients. The strongest positive correlation (*r* = 0.688) was observed between pyraclostrobin and azoxystrobin (Figure 2). Mefentrifluconazole had a moderate correlation with pyraclostrobin (*r* = 0.357) and a strong correlation with azoxystrobin (*r* = 0.556).

The EC_50_ values of the tested *P. tritici-repentis* strains varied in a wide range, from 0.076 mg L^−1^ to 12.425 mg L^−1^, depending on the active ingredient (Table 3). The lowest EC_50_ was determined against prothioconazole (0.091–4.080mg L^−1^), but the highest EC_50_ values were against mefentrifluconazole (1.663–12.425 mg L^−1^). Pyraclostrobin and prothioconazole had similar EC_50_ value ranges (0.076–4.462 mg L^−1^), while azoxystrobin had minimal EC_50_ value variation (0.351–2.929 mg L^−1^).

### 3.2. Detection of QoI Target Site Mutations

**Figure 3:**
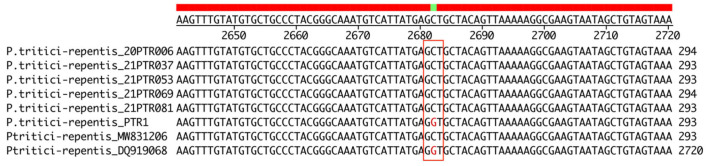
Clustal W nucleotide sequence alignment of cytochrome b gene fragment of *Pyrenophora tritici-repentis* and comparison with DQ919068 (wild type) and MW831206 (carrying G143 mutation). The target site of mutation G143A, where wild-type nucleotide triplet GGT is replaced by triplet GCT, is marked with a red rectangle.

Only one strain, PTR1, used in EC_50_ calculation experiments did not harbour a QoI target site mutation and had considerably lower EC_50_ values (Table 3). The rest of the screened strains carry the G143A mutation (Figure 3). Mutations F129L and G137R were not found in the screened strain subset.

## 4. Discussion

Since the first sightings of tan spot in the late 1990s, it has become the dominant wheat disease in Latvia. Similarly, as in other wheat-growing regions, Latvian farmers mainly rely on fungicide sprayings to control wheat diseases. *Zymoseptoria tritici* (causal agent of Septoria leaf blotch) and *Puccinia striiformis* (causal agent of yellow rust) are comparatively well studied in regards to the fungicide field efficacy and target site mutations that affect pathogens’ sensitivity to fungicides [18,31,32]. There are almost no data about *Pyrenophora tritici-repentis* sensitivity to various fungicides in Latvia and surrounding countries. Only a few studies have looked at the inhibition effect of various fungicide a.i.’s on the pathogen’s mycelium growth in Europe, Australia, Canada and Argentina [20,21,33,34].

In the present study, we demonstrated the variation in sensitivity to the most commonly used DMI and QoI fungicides on the Latvian *P. tritici-repentis* population. The fungicide performance was mainly determined by each a.i., showcased by differences in strain sensitivity to substances in the same mode of action. *P. tritici-repentis* strains showed a large variance in sensitivity to the fungicides’ a.i.’s, except for the prothioconazole. Complementary data have been observed in Estonia, where *Pyrenophora teres* f. *teres* showed more uniform response to prothioconazole and mefentrifluconazole compared to a.i. form succinate dehydrogenase inhibitors (SDHIs) and quinone outside inhibitors (QoIs) [35]. The authors have found that pathogen sensitivity to fungicides fluctuates between years, which in turn could also explain the observed variation in this experiment [35,36].

There were no indications that sensitivity to fungicides is related to the isolate origin, indicating that the *P. tritici-repentis* population is probably not segregated by the host. Similar results between host plant and fungicide resistance were detected also in *Botrytis cinerea* strains isolated from various horticultural crops in Germany [37], but there are limited data about host plant influence on pathogen fungicide resistance.

In general, *P. tritici-repentis* was more sensitive to pyraclostrobin than to azoxystrobin, which is supported by other studies [21,38,39]. EC_50_ values to pyraclostrobin observed in this study were multiple times higher than reported in North America [25,34]. These data indicate that the *P. tritici-repentis* population in Latvia has seen rapid decrease in sensitivity, which could be explained by the lack of fungicide resistance management practises among farmers. Strain sensitivity to azoxystrobin was within the range of the previous report by Sierotzki et al. [20], where EC_50_ values of wild-type strains varied from 0.007 to 0.725 mg L^−1^ and 0.71 to >100 mg L^−1^ for strains with the G143A mutation.

Prothioconazole was the most effective a.i. in this study. Similar results were reported by Tonin et al. [40], where prothioconazole showed the best efficacy in *P. tritici-repentis* mycelium growth inhibition, outperforming epoxiconazole and tebuconazole. Data from Australia have shown *P. tritici-repentis* sensitivity to epoxiconazole, propiconazole and tebuconazole, with EC_50_ values within the range up to 1 mg L^−1^ [33]. High sensitivity to propiconazole has also been reported in Canada, where EC_50_ values for the most tested strains varied from 0 to 0.9 mg L^−1^ [34].

The median inhibition of mycelial linear growth by mefentrifluconazole was only 11.11%, and EC_50_ values reached 15.471 mg L^−1^, suggesting that mefentrifluconazole is less suited for the tan spot control in Latvia in comparison to prothioconazole. Related species of *P. tritici-repentis*, e.g., *P. teres* f. *teres*, are less sensitive to mefentrifluconazole in comparison to prothioconazole-desthio [36]. However, mefentrifluconazole was highly effective in controlling other leaf pathogens, such as *Zymoseptoria tritici*, which is the cause of one of the most important wheat leaf diseases: septoria leaf blotch. It has shown high effectivity in vitro against triazole-resistant strains of *Z. tritici* [41,42]. While in other pathogens, like *Alternaria alternata* species complex, *Cercospora beticola* and *Colletotrichum* species, in vitro efficacy of mefentrifluconazole is either equal or slightly weaker compared to propiconazole, difenoconazole and tebuconazole [43].

Strains from the Czech Republic, 12015JP, 12016JP and 11020JP, were considerably more sensitive to azoxystrobin, pyraclostrobin and mefentrifluconazole than the screened strains from Latvia. This could be explained by the fact that the strains from Latvia are isolated more recently from the population exposed to a different fungicide application history. The only exception was sensitivity to prothioconazole, where strain reaction to this a.i. was uniform. The sensitivity of these strains to pyraclostrobin and azoxystrobin suggests that they are wild-type, with no QoI target site mutations. The sensitivity of 12015JP, 12016JP and 11020JP (Supplementary Table S1) to mefentrifluconazole, while the rest of the screened strains from other countries were insensitive, indicates that some unknown factors may determine strain sensitivity to this a.i.

The observed strong positive correlation between pyraclostrobin and azoxystrobin inhibition patterns can be explained by the fact that both a.i.’s have the same mode of action and are subject to the same three known target site mutations that grant cross-resistance [17]. Our study confirmed that the G143A mutation, which confers significant insensitivity to QoI fungicides, is present in the *P. tritici-repentis* population in Latvia, with the exception of the wild strain PTR1 from 2003. The signs of resistance formation to QoI fungicides have also been observed in the Fungicide Resistance Action Committee (FRAC) monitoring programmes [22]. The strong positive correlation between strain reaction to azoxystrobin and mefentrifluconazole was likely due to their weak effect on *P. tritici-repentis* mycelial growth inhibition.

## 5. Conclusions

This study provided insight into Latvia’s *P. tritici-repentis* population’s sensitivity to active ingredients of DMI and OoI fungicide groups, helping to fill the knowledge gap about the pathogen fungicide sensitivity in this region. Currently, prothioconazole-based products are advised for tan spot control in Latvia. However, this poses severe threats to sustainable tan spot control and *P. tritici-repentis* resistance risk management in the future.

## Figures and Tables

**Table 1:** Summary of *Pyrenophora tritici-repentis* strains included in the study.

Collection Year	Host		Country of Origin	Number of Strains
	Common Name	Scientific Name		
1970s	wheat	*Triticum aestivum*	Canada	2
1998	wheat	*Triticum aestivum*	USA	2
2001	wheat	*Triticum aestivum*	Czech Republic	1
2002	wheat	*Triticum aestivum*	Czech Republic	1
2003	wheat	*Triticum aestivum*	Latvia	3
2010	wheat	*Triticum aestivum*	Latvia	3
2010	wheat	*Triticum aestivum*	Finland	1
2011	wheat	*Triticum aestivum*	Czech Republic	2
2012	wheat	*Triticum aestivum*	Czech Republic	2
2019	wheat	*Triticum aestivum*	Latvia	12
2020	couch grass	*Elymus repens*	Latvia	2
2020	orchard grass	*Dactylis glomerata*	Latvia	2
2020	triticale	*xTriticosecale*	Latvia	1
2021	perennial ryegrass	*Lolium perenne*	Latvia	1
2021	triticale	*xTriticosecale*	Latvia	1
2021	orchard grass	*Dactylis glomerata*	Latvia	1
2021	meadow fescue	*Festuca pratensis*	Latvia	1
2021	red fescue	*Festuca rubra*	Latvia	1
2021	couch grass	*Elymus repens*	Latvia	1
2021	wheat	*Triticum aestivum*	Latvia	34
2022	spelt wheat	*Triticum spelta*	Latvia	18
2022	wheat	*Triticum aestivum*	Lithuania	1
Total				93

**Table 2:** *Pyrenophora tritici-repentis* strains used for EC_50_ value estimation.

Collection Year	Strain	Country of Origin	Host
2003	PTR1	Latvia	*Triticum aestivum*
2020	20PTR006		*Elymus repens*
2021	21PTR069		*Dactylis glomerata*
	21PTR053		*Triticum aestivum*
	21PTR037		*Lollium perenne*
	21PTR081		*Festuca rubra*

**Table 3:** The effective concentration (EC_50_) values of *Pyrenophora tritici-repentis* strains to fungicide active ingredients.

Active Ingredient	Strain	EC_50_ Value, mg L^−1^	Standard Error
pyraclostrobin	PTR1	0.076	0.015
	21PTR069	1.140	0.205
	21PTR053	1.140	0.219
	20PTR006	1.228	0.246
	21PTR037	3.521	0.529
	21PTR081	4.462	1.083
azoxystrobin	PTR1	0.351	0.055
	21PTR037	0.893	0.046
	21PTR081	1.061	0.064
	20PTR006	1.912	0.117
	21PTR069	2.028	0.092
	21PTR053	2.235	0.109
prothioconazole	21PTR037	0.091	0.016
	21PTR081	0.139	0.013
	21PTR069	0.186	0.015
	20PTR006	0.201	0.012
	21PTR053	0.310	0.030
	PTR1	0.980	0.232
mefentrifluconazole	21PTR081	1.663	0.127
	21PTR037	2.086	0.313
	21PTR069	3.557	0.354
	20PTR006	5.132	0.421
	21PTR053	6.920	0.370
	PTR1	12.425	0.679

## Data Availability

The data presented in this study are available on request from the corresponding author.

## Supplementary Materials

The following supporting information can be downloaded at https://www.mdpi.com/article/10.3390/pathogens13121060/s1; Supplementary Table S1: Origin of *Pyrenophora tritici-repentis* isolates.
